# Climate change and mental health: an overview of UK policy and regulatory frameworks to stimulate and inform future research and practice

**DOI:** 10.1192/bjp.2024.216

**Published:** 2024-11-25

**Authors:** Andrea Mechelli, Lea Baecker, Ioannis Bakolis, Robert Stewart, Antonio Gasparrini, Michael Smythe, Matthew White, Nick Bridge

**Affiliations:** Department of Psychosis Studies, Institute of Psychiatry, Psychology & Neuroscience, King’s College London, De Crespigny Park, London SE5 8AF, UK; Health Service and Population Research Department and Department of Biostatistics and Health Informatics, Institute of Psychiatry, Psychology and Neuroscience, King’s College London, De Crespigny Park, London SE5 8AF, UK; Department of Psychological Medicine, Institute of Psychiatry, Psychology and Neuroscience, King’s College London, De Crespigny Park, London SE5 8AF, UK; Environment & Health Modelling (EHM) Lab, Department of Public Health Environments and Society, London School of Hygiene & Tropical Medicine, London WC1H 9SH, UK; Nomad Projects, Sunbury Workshops, 24, Swanfield St, London E2 7LF, UK; Herbert Smith Freehills LLP, Exchange House, Primrose Street, London EC2A 2EG, UK; Policy Advisor, King’s College London, De Crespigny Park, London, SE5 8AF, UK

## Abstract

In the context of climate change, the impacts of extreme weather events are increasingly recognised as a significant threat to mental health in the UK. As clinicians and researchers with an interest in mental health, we have a collective responsibility to help understand and mitigate these impacts. To achieve this, however, it is vital to have an appreciation of the relevant policy and regulatory frameworks. In this feature article, a collaboration amongst mental health and policy experts, we provide an overview of the integration of mental health within current climate policies and regulations in the UK, including gaps and opportunities. We argue that current policy and regulatory frameworks are lacking in coverage, ambition, detail and implementation, as increases in weather extremes and their negative impacts on mental health outpace action. For example, across current national and local climate policies, there is almost no reference to the impacts of extreme weather events on mental health. Whilst alarming, this provides scope for future research to fill evidence gaps and inform policy and regulatory change. We call for mental health and policy experts to work together to improve our understanding of underlying mechanisms and develop practical interventions, helping to bring mental health within climate policy and regulatory frameworks.

## Introduction

The climate crisis is a public health crisis. In addition to the well-established physical harm, increasing frequency and intensity of extreme weather events, such as heatwaves, floods and wildfires, is posing a significant threat to the mental health of affected communities. While in the past the impacts of these events have been felt mostly in the Global South, in recent years extreme weather events have become a common occurrence in places with milder climate such as the United Kingdom (UK).

A recent report from the Meteorological Office highlights that the UK is experiencing more frequent and intense heatwaves, with 40°C being recorded for the first time in several locations in 2022.^1^ Temperatures at this exceptional level were not isolated events but reflect an accelerated trend in the UK, where all of the 10 hottest recorded years between 1884 and 2022 have occurred during the 21st century. However, the impacts of climate change are not limited to increases in ambient temperature, as the UK is also experiencing a dangerous combination of heavier rainfalls and longer spells of drought.^1^

Climate change is increasingly recognised as a significant threat to mental health; for example, a recent meta-analysis found that heatwaves, defined as daily maximum temperatures of at least 35°C for at least 3 days, are associated with a 9.7% increase in hospital attendance or admission for mental illness.^2^ The vast majority of research, however, has been carried out in countries such as the USA, Australia and China, where the impacts of extreme climate have long been recognised. This means that at present we have little understanding of how climate change is affecting the mental health of individuals and communities in the UK. As researchers and clinicians with a focus on mental health, we have a collective responsibility to help understand and mitigate these impacts.^3^ To achieve this, it is vital to have an appreciation of the current policy and regulatory frameworks in this area.

In this feature article, a collaboration between mental health and climate policy experts and community leaders, we provide an overview of the integration of mental health within current climate policy and regulation in the UK, including gaps and opportunities. We show that current policy and regulatory frameworks are lacking in coverage, ambition, detail and implementation, as increases in weather extremes and their negative impacts on mental health outpace action. Whilst alarming, this provides scope for future research to fill evidence gaps at both national and local levels.

### Current climate policies and regulations in the United Kingdom

**Figure 1:**
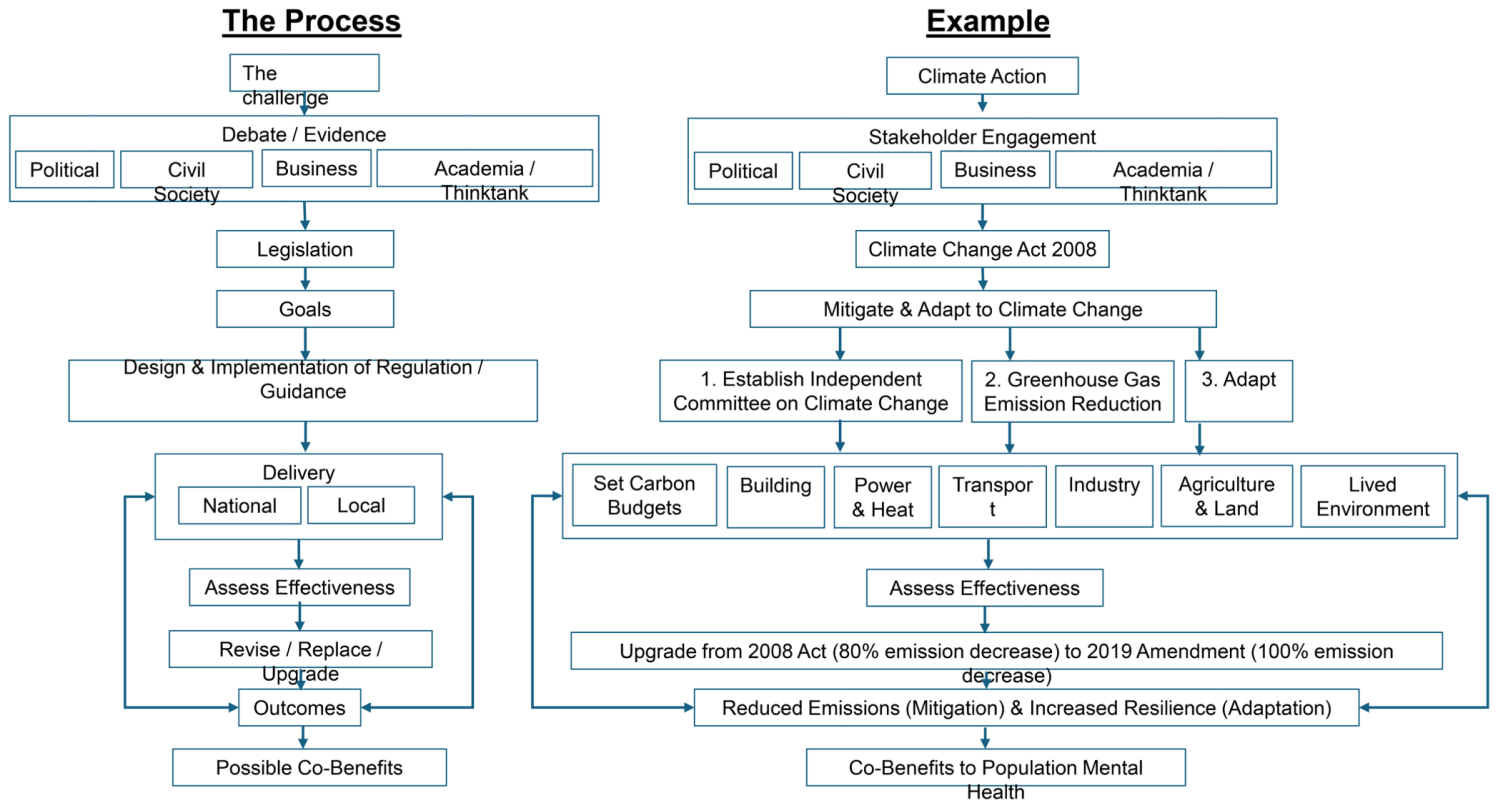
The policy process (left) and illustrative example (right).

The UK has adopted a range of instruments at national and sub-national level that aim to mitigate climate change and its impacts. Headline goals and targets are set by national statutory legislation enacted by the Parliament,^4^ against which the Government is legally held to account. However, outcomes under national statutory legislation are a blend of different policy actions, requiring substantial cooperation across national and sub-national authorities and economic sectors. A schematic representation of the policy process in the UK is provided in Figure 1.

A summary of key policies, regulations and guidance with direct or indirect bearing on climate change action in the UK can be found in Table 1. At the harder end of the spectrum are legally-enforceable policies and regulations, such as planning laws; through to frameworks and guidance, such as the National Planning Policy Framework, which can also carry significant legal weight in planning decisions; to the softer end of the spectrum which includes more market-led or voluntary measures, such as advisory services, voluntary codes of practice, and cooperation programmes such as the Local Net Zero Hubs. Successful societal outcomes tend to rely on a blend of all of these measures.

#### National policies

The Climate Change Act 2008^4^ sets the overarching national framework for mitigating and adapting to climate change in the UK. Under this Act, the UK is legally required to reduce greenhouse gas emissions to at least 100% below 1990 levels by 2050 (i.e., "net zero") and to set out objectives in relation to adaptation to climate change, together with proposals, policies and timescales for meeting those objectives.

In practice, in terms of climate mitigation (i.e. the process of reducing the emission of greenhouse gases), this involves meeting five-yearly Carbon Budgets, which achieve progressive reductions in emissions until ‘net zero’ emissions by 2050. Measures to achieve this goal are set out in the Government’s Net Zero Strategy 2021, upgraded to the Net Zero Growth Plan & associated policies in 2023, with policy details captured in the Carbon Budget Delivery Plan. However, while these documents mention the goal of supporting health and wellbeing, there is no explicit reference to mental health. Meanwhile, in terms of adaptation (i.e. the process of adjusting to current and/or projected impacts of climate change), this involves the production of a five-yearly climate change risk assessment to identify risks, and a National Adaptation Programme identifying measures to address these risks. The Government has published the third National Adaptation Programme (NAP3), which sets out actions that the Government and others will take to adapt to the impacts of climate change up to 2028. These include increasing the resilience of transport and building infrastructure to counteract increasing temperature extremes, investing in the natural environment, and strengthening agricultural resilience to protect public health. However, while NAP3 mentions the importance of supporting public health, there is no explicit reference to mental health.

Another policy instrument with significant implications for climate change action is the National Planning Policy Framework (NPPF), which includes policies relevant to infrastructure, housing, and economic development, all of which are determinants of mental health and healthcare delivery systems. The NPPF highlights the need to manage the risk of overheating from rising temperatures and the importance of supporting the future resilience of communities when planning for and building new developments.^5^ A number of practical suggestions are made, including the expansion of green infrastructure and the use of natural ventilation in buildings. Within the NPPF there is a reference to solutions that support and promote “healthy lifestyles”, however once again there is no explicit reference to mental health.

A number of additional policies provide guidance on standards for supporting infrastructure while protecting the natural environment, such as the National Infrastructure Assessment published by the National Infrastructure Commission. However, mental health is not explicitly considered within these policies.

In brief, there are no measures that explicitly address mental health in the Climate Change Act 2008 or in any other national mitigation and adaptation policies. This is the case despite the fact that the implementation of these policies (or the lack of it) will have direct and indirect implications for the mental health of individuals and communities in the UK.

#### Local policies

Local Authorities are being proactive in tackling climate change: in recent years 95% of Local Authorities have declared climate emergencies, and two thirds have pledged to be carbon neutral by 2030, twenty years ahead of the national target date.

Local-level action to reduce emissions stems from national policy frameworks, such as the Climate Change Act 2008, in areas like implementation of energy networks, investment in low carbon public transport, energy efficiency standards, planning regulations for new buildings, retrofitting and support to businesses and constituents. The Government supports a number of local initiatives, including the Local Net Zero Accelerator Programme, Local Net Zero Hubs and Community Energy support programmes. The Local Authority Association for England & Wales also has a range of partnerships for delivering net zero goals. Local Authorities have a key role in helping communities adapt to climate change, including protecting public health, providing green and blue public spaces, and maintaining physical infrastructure.^6^ However, similar to the national level, there is little or no explicit reference to mental health in current Local Authority climate action plans.

Considering one example of local policy, London has one of the most ambitious action plans to tackle climate change in the world. Key environmental and sustainability ambitions are set out in the London Plan 2021, a spatial development plan which runs to 2041. The London Plan mostly contains non-specific, broad objectives. On extreme heat specifically, it proposes to reduce outdoor temperature in urban heat islands and mitigate internal heat gain via design, ventilation and materials. However, as with the NPPF, these heat management and cooling hierarchies are not mandatory and the Mayor of London only has the opportunity to enforce London Plan policies directly in relation to larger projects that are of strategic significance to London as a whole.

There are some references in the London Plan to mental health, but they are not linked to climate impacts such as extreme heat. For example Chapter 8, which focuses on Green Infrastructure, mentions climate adaptation, the urban heat island effect and mental health, however there are no explicit links between them. This highlights the current lack of a joined-up approach when considering climate extremes and mental health, despite the multiple links among them. In May 2024, Sadiq Khan was re-elected as Mayor of London for a third term. The environment will remain a central component of his programme, and a new London Plan is expected to be published by March 2026.

In brief, local authorities and cities play a critical role in tackling climate change as a result of their democratic, policy and financial responsibilities, assets and relationships. Current policies and regulations at the local level aimed at protecting the environment and/or mitigating climate impacts do not explicitly consider or address mental health outcomes. However, many of these initiatives will have direct and indirect implications for the mental health of affected individuals and communities.

#### International policies

While this article focuses on the policy environment in the UK, it is important to consider wider global context. While the UK is relatively well-placed in terms of its financial, human and policy resources, there is much to learn from the experience of other countries. Examples include Burkino Faso’s use of natural local materials and traditional design for resilient public buildings, Paris’ successful planning and implementation of the 15-minute city concept, and Copenhagen’s goal of carbon neutrality by 2025 through energy production and low emissions mobility. In addition, the UK could benefit from engaging with global initiatives aimed at promoting and supporting the integration of mental resilience with climate resilience, such as the recent roadmap for care and change^7^ co-developed by a diverse network of more than 450 organisations. Many of these initiatives were developed to support the wellbeing of local communities in the face of climate extremes, and as such provide practical opportunities for exchange of best practice which could be integrated in future policies and regulations within the UK.

### Opportunities to shape the national and local political agenda

There are several opportunities for mental health researchers and clinicians to contribute to the emerging evidence on climate change and mental health in the UK, helping inform and shape the national and local political agenda.

#### Healthcare protocols to reduce mental health risks

At present, across the healthcare sector, there are no formal protocols for reducing mental health risks in the context of extreme heat or other extreme weather events. The development and validation of such protocols could help mitigate the mental health impacts of these events in the future. These protocols might include, for example, better use of data to inform the allocation of resources, early warning systems to increase resilience and proactive support to people who may be particularly vulnerable to extreme events, such as those with pre-existing mental health conditions. These protocols would need to be developed and validated in consultation with stakeholders including NHS England, local Clinical Commissioning Groups, Health and Wellbeing Boards, Sustainability and Transformation Partnerships and Integrated Care Systems. Target policies: Adverse Weather and Health Plan, Health & Security Agency agenda, National Institute for Health & Care Research (NIHR) Health Protection Research Unit agenda.

#### The mental health co-benefits of net zero or climate-positive national building codes, materials & design

Heating and powering buildings accounts for 30% of the total energy usage in the UK. Therefore, the retrofitting of existing buildings and construction of zero carbon and resource-efficient buildings, through a radical overhaul of building regulations, will be critical to net zero targets. The potential mental health co-benefits of these initiatives for the affected communities are not well understood and are not considered within current policies and regulations. A better understanding and recognition of these co-benefits could accelerate and incentivise net zero and climate-positive national building codes, materials & design, for instance through targeted public procurement and the use of industry-leading embedded carbon and other standards. Target policies: Future Homes Standard, Building Regulations, National Development Management Policies.

#### Reductions in air and noise pollution in transport plans

Car traffic is a major source of air^8^ and noise^9^ pollution, both of which have been associated with significant increases in mental health risk. Local initiatives to reduce car traffic, such as the London’s Ultra-Low Emission Zone (ULEZ) in London, have been met with mixed response by citizens and businesses, resulting in a number of protests and campaigns in the affected areas. As the possible introduction, reduction or expansion of low emission zones is being debated across the UK, and wider efforts are made to accelerate the introduction of more sustainable, cleaner and quieter transport systems, there is an urgent need for more research on the co-benefits of these initiatives for mental health to help provide a robust evidence base for future decision-making. Target policies: National Planning Policy Framework, Planning Practice Guidance, the London Plan and London Plan Guidance, London Environment Strategy 2018, Borough local plans and supplementary planning documents.

#### Natural ecosystems and biodiversity in urban areas

Natural ecosystems and biodiversity are a central defence against climate change but also important pillars of mental health, especially for those who live in cities.^10^ The risk of developing two of the most prevalent mental disorders in the world, depression and anxiety, is lower in urban dwellers who spend time in green spaces compared to those who do not.^11^ Furthermore, even brief incidental encounters with nature as part of everyday life can have a positive impact on mental wellbeing which is still evident several hours later.^12^ The benefits of urban nature for mental health are explicitly recognised in a number of existing policies and regulations for protecting and enhancing natural environments in the UK, such as the Environment Act 2021 and the 25 Year Environment Plan. In addition, planning obligations under section 106 of the Town and Country Planning Act 1990 require new private sector developments to provide publicly-accessible green spaces in urban areas. However, not all green spaces have the same benefits. For example, green spaces with higher levels of biodiversity are associated with greater mental health improvements than those with lower levels.^13–15^ Yet current policies and regulations refer to nature in generic terms and lack specific guidance on how to maximise benefits. A better understanding of the mechanisms which underlie the relationship between nature and mental health, would help make future policies and regulations in this area more actionable and impactful. Target policies: Environmental Improvement Plan 2023, Biodiversity Net Gain, National Planning Policy Framework, Planning Practice Guidance, London Environment Strategy 2018, London Plan and London Plan Guidance, Borough Open Space and Biodiversity Strategies, Borough local plans and supplementary planning documents, Borough Green Infrastructure Plans, Mental Health and Town Planning RTPI Practice Advice.

#### Acceleration of specific proposals for increasing green infrastructure in London and other cities

A number of local authorities are developing plans to increase biodiversity, green and blue spaces, shading and natural cooling initiatives. However, the implementation of these plans can be hindered by resistance to change and financial constraints. There is an opportunity for mental health research to evaluate the health co-benefits of these initiatives, for example by quantifying mental health impacts in the affected communities and how these impacts translate into economic benefits for the wider society. Target policies: London Environment Strategy 2018, The London Plan.

#### City-wide heating & cooling plan through adoption of smart surfaces/buildings

Local authorities are considering the adoption of smart surfaces/buildings (e.g. permeable, reflective, cooling, energy generating) for all new build and upgrading of existing infrastructure such as road and pavement re-surfacing and upgrades, roofing materials and solar photovoltaic, especially in public buildings such as schools, prisons, social housing and airports. Such measures can significantly reduce temperatures and use of fossil-fuel driven air conditioning, which can be costly for poor households, displaces rather than removes heat, and increases emissions. A better understanding of mental health impacts, including benefits and risks, could strengthen the evidence base of these initiatives. Target policies: Building Regulations, Borough local plans and supplementary planning documents, public procurement policies and standards (e.g. Carbon Star).

#### The 15-minute city

A number of local authorities are taking steps towards the planning and implementation of the 15-minute city concept, or variants of this community-focused approach, where citizens are able to meet most, if not all, of their needs within a short walk or bike ride from their home. While the concept was developed to reduce carbon emissions by decreasing the use of cars, its implementation is likely to have measurable impacts on the mental health of affected communities through a number of mechanisms. These might include, for example, higher levels of physical activity and socialization, lower levels of air and noise pollution, possible mitigation of the urban heat island effect, more time spent in contact with nature. In light of the ongoing debate and controversy around the 15-minute city concept^16^, it would be important to assess possible mental health impacts in local communities and how these interact with existing sociodemographic factors (e.g. indices of multiple deprivation). Target policies: London Plan and London Plan Guidance, London Environment Strategy 2018, Borough local plans and supplementary planning documents, Mental Health and Town Planning RTPI Practice Advice.

#### Assessing international climate actions through a global mental health lens

There are numerous channels for international collaboration towards the transition to net zero, for example COP26 Breakthrough Coalitions (in industry, transport, energy, cement, agriculture, chemicals), OECD best practice committees, Mission Innovation, World Economic Forum, World Business Council for Sustainable Development, Race to Zero^17^, the C40 network^18^ and the Global Covenant of Mayors for Climate & Energy^19^. In the vast majority of cases, these channels promote actions to reduce greenhouse emissions without explicitly considering mental health impacts. This means the wider co-benefits of these actions, such as financial savings to the healthcare sector and increased health and productivity of vulnerable workers, are not adequately recognised. There is scope for mental health researchers to help inform and assess these ongoing initiatives through a global mental health lens. Target policies: UNFCCC policy and guidance, UN Sustainable Development Goals.

## Discussion

We have shown that the impacts of climate change on mental health are not adequately considered in existing climate policies at national or local level. Current initiatives fail to recognise that aspects of our everyday environment such as air quality, ambient temperature, access to green and blue space and biodiversity are critical determinants of population mental health. However, even without explicit recognition, current mitigation and adaptation policies are likely to be hugely beneficial for the mental health of the population. Examples include the ULEZ to stimulate active travel and reduce air and noise pollution in London, the recent Biodiversity Net Gain legislation (*Environment Act 2021*^20^) to promote a greener built environment in England, the Environmental Improvement Plan 2023 to support the creation of 15-minute cities and the Adverse Weather and Health Plan^21^ to trigger actions in the NHS. A comprehensive evaluation of these policies through a mental health lens, considering not only clinical outcomes but also wider societal outcomes, is important for at least three reasons.

Firstly, several policies to tackle climate change have been received with resistance from some stakeholders; a notable example is the introduction of the ULEZ in London, which has led to street protests, organised campaigns and misinformation. A better understanding and communication of the potential mental health co-benefits of these policies may strengthen their rationale and help alleviate concerns.

Secondly, a deeper understanding of the mental health impacts of climate policies would be important for optimising these impacts in future legislation, policy and regulation. For example, further research into the mental health consequences of existing legislation to adapt transport and building infrastructure to extreme heat in our cities could inform the next iteration of policies. Further examples of research priorities to inform future legislation can be found in the global^22^ and regional^23^ agendas for climate change and mental health research and action recently published by the Connecting Climate Minds network.

Thirdly, it is possible that some of the policies aimed at protecting the environment and addressing global warming might in fact have negative consequences for mental health^24^. For example, certain policies could lead to green gentrification and/or exacerbate social inequalities with detrimental impacts on the mental health of the affected communities. There is a need to understand and address such unintended consequences through a comprehensive evaluation.

In addition, our overview highlights the complexity of the policy environment and the need for close cooperation between different levels of government, and across departments, budgets, geographies and economic sectors. This complexity will need to be reckoned with when deciding on the most effective ways of delivering research and influencing policy. For example, the success of local initiatives is highly dependent on the national government having a clear vision and providing the financial resources to implement local plans. Another example is the current lack of cross-disciplinary cooperation across planning policy, urbanicity and public mental health, despite the clear linkages between these disciplines as highlighted by the emerging field of ‘neurourbanism’^25^.

While this might feel overwhelming to a mental health researcher who approaches these issues for the first time, a useful first step is to reach out to stakeholders who can help design and implement impactful research (Table 2). Here the relevant stakeholders will depend on the aims of the research; whether the focus is on the mental health of the general population or specific vulnerable communities will have a significant bearing on the identification of relevant stakeholders and the optimal strategy for policy engagement.

It is also important to make a distinction between existing policies and regulations on one hand, and actual implementation on the other hand. In 2008, the government established an independent advisor, the Committee on Climate Change (CCC), to oversee and advise on the progress made so far. In a recent assessment, the CCC concluded that the Government’s implementation is falling short across the board, including lack of prioritisation, funding and monitoring.^26^ This lack of progress at national level is impacting sub-national level action, where 95% of local authorities have declared climate emergencies but lacking the required resources and support from national government to implement ambitious plans.^27^ This makes the active participation of mental health experts in evaluating and communicating the mental health benefits of climate action all the more important.

In conclusion, in regard to the mental health impacts of climate change, UK climate policy and regulatory frameworks are lacking in coverage, ambition, detail and implementation. Whilst alarming, this provides scope for future research to fill evidence gaps and inform policy and regulatory change, helping address determinants of mental health and reduce inequalities through local, national and global mobilisation of efforts. The urgency of this is increasingly recognised by funding bodies, resulting in a proliferation of relevant funding initiatives in recent years (e.g. Wellcome’s Climate Impacts Award). We call for mental health researchers and clinicians to work together with climate policy experts and people with lived experience to improve our understanding of underlying mechanisms and develop practical interventions, helping bring mental health within climate policy and regulatory frameworks. A further consideration is that integrating mental health within climate policy is not enough, we also need to consider ways of effectively integrating climate change within mental health policy. While UK-focused, these actions will also be of relevance to other countries experiencing extreme weather events globally.

## Figures and Tables

**Table 1:** Summary table of key policies, regulations and guidance with direct or indirect relevance to climate change action in the UK. * weak strength measure, comprising guidance or best practice advice; ** medium strength measure, a mixture of legal measures and guidance; *** strong measure, underpinned by statutory legal powers.

National measures	Type	Next revision / update
		
Climate Change Act 2008	***	No plan for update
Climate Change Act 2008 (2050 Target Amendment) Order 2019	***	No plan for update
Planning Act 2008	***	No plan for update
HMG Carbon Budget Delivery Plan, 2023	***	Before May 2025
Adverse Weather and Health Plan, UK Health Security Agency	*	At any time
National Planning Policy Framework	**	At any time (last update December 2023)
Planning Practice Guidance	**	At any time (relevant sections last update 7/2019)
National Adaptation Programme	**	At any time (last update July 2023)
Environment Act 2021	***	No plan for update
National Infrastructure Commission, 2^nd^ assessment	*	At any time (last update November 2021)
Environmental Improvement Plan 2023	**	No plan for update
Biodiversity Net Gain	***	No plan for update (mandatory from 2/2024)
Building Regulations	***	At any time (last update October 2023)
National Development Management Policies	**	To be decided
		
**Local measures**		
		
Borough Local Plans and Supplementary Planning Documents	**	At any time
Borough Green Infrastructure Plans	**	At any time
Borough Open Space and Biodiversity Strategies	**	At any time
Local Design Codes	**	At any time
RTPI / TCPA Joint Guidance for Local Authorities on Planning for Climate Change	*	At any time
Local Net Zero Hubs programme	*	At any time
Local Net Zero Accelerator programme	*	At any time
Mental Health and Town Planning RTPI Practice Advice	*	At any time
London Climate Resilience Interim Review	*	Final report due mid 2024
The London Plan and London Plan Guidance	**	Focused review directed by September 2024, new plan not likely to be before mid-2026 (relevant sections last updated in 2023)
London Environment Strategy 2018	***	Latest Progress Report published March 2024
		
**International measures**		
		
UNFCCC policy and guidance	**	Reviewed annually at end of year
UN Sustainable Development Goals	*	2030

**Table 2:** Non-exhaustive list of potential stakeholders when developing and implementing research into climate change, mental health and climate policies.

Mayor’s Office and GLA London Plan teams London Assembly committees/members London Climate Change Partnership Local Planning Authorities Non-Government Organizations, e.g. Natural England, Forestry Commission, Parks for London, Arboricultural Association, Woodland Trust Transport for London Community & voluntary groups Commissioners of health or social care services in Local Authorities Directors of Public Health in Integrated Care Systems Providers of health or social care, e.g. General Practitioners, Primary and Community Healthcare, Hospitals, Adult Social Care, Children’s Social Care National government – Environment Agency, NHS England, UK Health Security Agency, Department of Health and Social Care, Met Office, Local Government Association Professional associations, e.g. Royal Town Planning Institute, Royal Institution of Chartered Surveyors, Town and Country *Planning* Association Real estate developers, consultants and advisers General public

## Data Availability

Data availability is not applicable to this article as no new data were created or analysed.
